# Carotid smooth muscle contractility changes after severe burn

**DOI:** 10.1038/s41598-021-97732-3

**Published:** 2021-09-10

**Authors:** Kevin DeSpain, Charles R. Rosenfeld, Ryan Huebinger, Xiaofu Wang, Jayson W. Jay, Ravi S. Radhakrishnan, Steven E. Wolf, Juquan Song

**Affiliations:** 1 Department of Kinesiology, University of Texas Arlington, Arlington, TX USA; 2 Department of Pediatrics, UT Southwestern Medical Center, Dallas, TX USA; 3 Department of Surgery, Shriners Hospitals for Children - Galveston, Galveston, TX USA; 4 Department of Surgery, University of Texas Medical Branch, 301 University Blvd., Galveston, TX 77555-0644 USA

**Keywords:** Molecular biology, Physiology, Diseases

## Abstract

Severe burns result in cardiovascular dysfunction, but responses in the peripheral vasculature are unclear. We hypothesize that severe burns disturb arterial contractility through acute changes in adrenergic and cholinergic receptor function. To address this, we investigated the changes in carotid artery contractility and relaxation following a severe burn. Thirty-four adult Sprague–Dawley male rats received a 40% total body surface area (TBSA) scald burn and fluid resuscitation using the Parkland formula. Control animals received sham burn procedure. Animals were serially euthanized between 6 h and 14 days after burn and endothelium-intact common carotid arteries were used for ex vivo force/relaxation measurements. At 6 h after burn, carotid arteries from burned animals demonstrated a > 50% decrease in cumulative dose-responses to norepinephrine (*p* < 0.05) and to 10^−7^ M angiotensin II (*p* < 0.05). Notably, pre-constricted carotid arteries also demonstrated reduced relaxation responses to acetylcholine (*p* < 0.05) 6 h after burn, but not to sodium nitroprusside. Histologic examination of cross-sectional planes revealed significant increases in carotid artery wall thickness in burned rats at 6 h versus 3 days, with increased collagen expression in tunica media at 3 days (*p* < 0.05). Carotid artery dysfunction occurs within 6 h after severe burn, demonstrating decreased sensitivity to adrenergic- and angiotensin II-induced vasoconstriction and acetylcholine-induced relaxation.

## Introduction

Severe burns result in organ dysfunction and changes in the cardiovascular system, which are accompanied by complications such as hypotension and tissue hypoperfusion^1^. Patients with greater than 15–20% total body surface area (TBSA) burn are at high risk of hypovolemic shock secondary to increased fluid losses in the absence of appropriate fluid resuscitation and cardiovascular compensation^2^. A response called ‘burn shock’ was previously identified in the first 24 h after injury and noted to peak at 6–8 h after burn^3^. Burn shock is thought to be due to decreased cardiac output and increased vascular resistance, though the effects on the peripheral vasculature are not clearly defined. Although cardiac dysfunction has been well studied in burned patients^4^, the influence of burn injury on the peripheral circulation has not been fully investigated.

Following severe burn, stress hormones, including catecholamines and cortisol, are released in order to drive subsequent pathophysiological and metabolic responses^5^. Along with other vasoactive mediators, serum epinephrine is dramatically increased in trauma patients within 4 h from injury^6^. These responses, when prolonged, lead to complications such as cardiac dysfunction and delayed wound healing^7^. However, what effect these changes have on peripheral arterial function is not known.

Vascular function is affected by interaction of endothelial and smooth muscle mediated mechanisms. Vasoconstriction and vasodilation contribute to regulation of segmental blood flow and are controlled by endogenous and exogenous signals^8^. Vasoconstriction is induced by changes in Ca^2+^, adenosine triphosphate (ATP), α1-adrenergic receptor agonists and angiotensin II (AngII) through activation of vascular smooth muscle (VSM) specific receptors and ion channels^9^. Vasodilation is regulated by β2-adrenergic receptor activation and acetylcholine stimulation of endothelial nitric oxide (NO) release. Thus, both endothelial-dependent and -independent pathways contribute to arterial vascular regulation and tone^10^.

In this study, we posit that severe burns alter peripheral arterial vascular contractility by modifying adrenergic and cholinergic receptor-mediated smooth muscle contraction. Therefore, the purpose of this study then was to determine the serial changes in the contractility profiles in carotid artery VSM following severe burn. We now report for the first time the dynamic changes that occur in carotid artery function following severe burn.

## Results

### Vasoconstrictor responses

**Figure 1:**
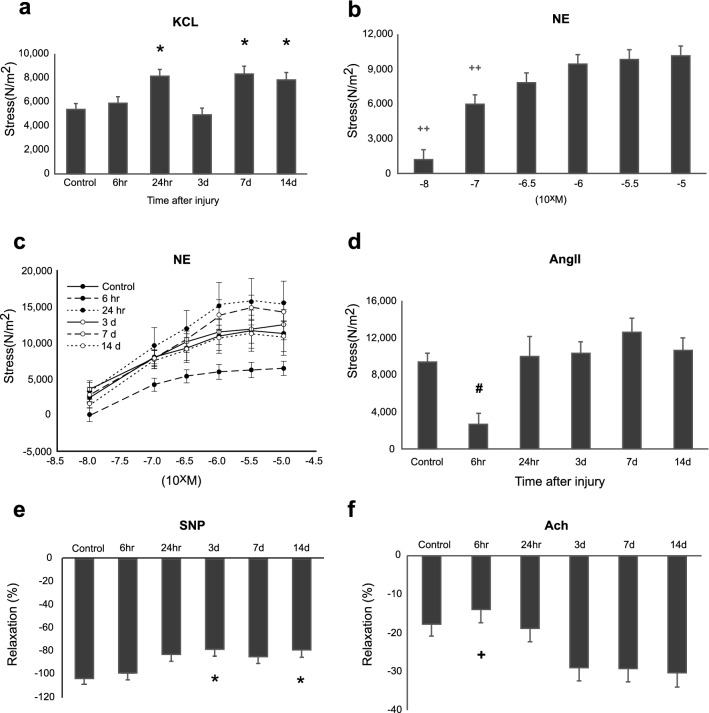
(**a**) Carotid smooth muscle contractility in burn rats with the cumulative dose of KCl time response. *, *p* < 0.05, versus control; (**b**) The cumulative dose response of rat carotid smooth muscle contractile in rats with norepinephrine (NE) stimulation. + +, *p* < 0.05, versus higher dose groups; (**c**) The effect of days after burn on the NE dose–response of carotid smooth muscle contractility. #, *p* < 0.05, versus other groups; (**d**) Carotid smooth muscle contractile alterations in burn rats with AngII stimulation. #, *p* < 0.05, versus others; (**e**) Precontracted carotid smooth muscle relaxation in burn rats with SNP stimulation, and (**f**) Ach stimulation over the time. *, *p* < 0.05, versus control; +, versus 3, 7, 14 days (**d**). The number of animals was 9, 7, 7, 7, 7 and 6 in the control, 6 h, 24 h, 3 days, 7 days, and 14 days group, respectively. Two-way analysis of variance (ANOVA) with post hoc Holm-Sidak method was applied. Data were presented as mean ± SEM.

KCl elicited dose-dependent nonreceptor-mediated contractions in endothelium-intact carotid arteries at each time point after burn (*p* < 0.001). When the maximum response at 65 mM KCl was analyzed across time, the responses were ~ 50% greater than controls and 6 h at 24 h, 7 days, and 14 days after burn (*p* ≤ 0.05) (Fig. 1a).

Norepinephrine (NE) also elicited dose-dependent contractions in intact carotid arteries (Fig. 1b; *p* < 0.001). The cumulative dose–response to NE at 6 h was significantly below all other time points (Fig. 1c; *p* < 0.001).

AngII is frequently associated with tachyphylaxis; thus, only one dose was examined in each carotid artery segment. At 6 h after burn, 10^–7^ M AngII showed a 70% decrease in stress generated contractions in intact carotid arteries compared to controls and all other time points (Fig. 1d, p < 0.001). Responses did not differ between controls, 24 h, 3 days, 7 days, and 14 days after burn.

### Vasodilator responses

After precontraction with NE 10^–5^ M, the endothelium-intact carotid arteries showed both dose and time-dependent relaxation responses to sodium nitroprusside, a nitric oxide (NO) donor (Fig. 1e,* p* ≤ 0.003). Furthermore, sodium nitroprusside elicited a significant relaxation at each dose (10^–8^ M–10^–6^ M) and plateaued at 10^–5^ M to 10^–6^ M, which did not differ. When each time point was compared, control carotid artery relaxation was greater at 3 days and 14 days (*p* ≤ 0.035), but did not differ at 6 h, 24 h, and 7 days.

Responses to sodium nitroprusside bypass the role of the endothelium-derived NO. Therefore, we assessed acetylcholine (Ach)-induced relaxation in intact arteries after precontracting with NE. We observed both a dose- and time-dependent relaxation response to acetylcholine (*p* < 0.001), demonstrating the presence of endothelium-derived NO. The response to Ach was maximum at 10^–5^ M. Among the time points examined, relaxation responses at 6 h were not different from control, but were significantly less than that at 3 days, 7 days, and 14 days (*p* ≤ 0.029; Fig. 1f).

### Histology assessment of carotid artery

**Figure 2:**
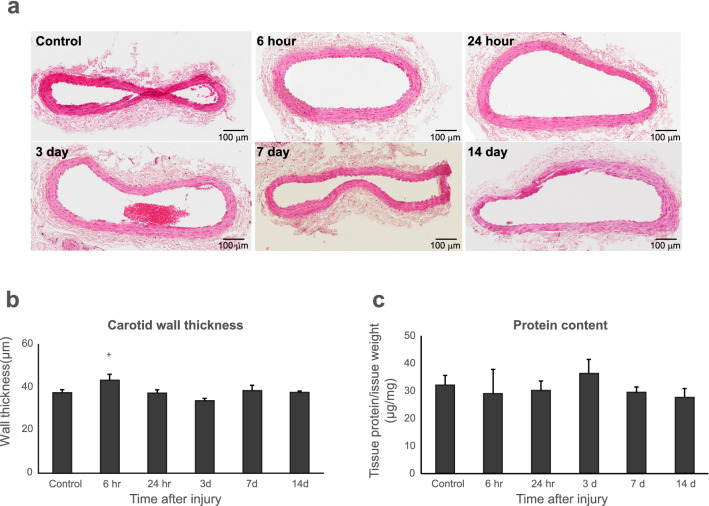
(**a**) Histology images of rat carotid ring tissue; (**b**) Statistical analysis of carotid wall thickness in burn rats; (**c**) Carotid protein content in burn rats. +, *p* < 0.05, 6 h (hr) versus 3 days (**d**). One-way ANOVA with Bonferroni t-tests was applied. Data were presented as mean ± SEM.

**Figure 3:**
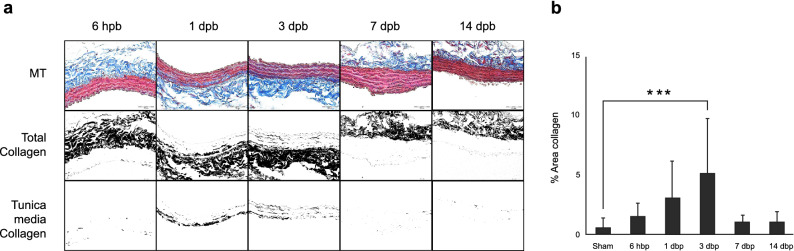
(**a**) Masson’s trichrome stain shows significantly increased collagen expression within the tunica media smooth muscle of the carotid artery at 1 day and 3 days after burn compared to sham-treated animals. Top row: Masson’s trichrome stain (MT); collagen fibers stain blue, muscle fibers stain red. Middle row: Color thresholding ImageJ method shows total collagen in black. Bottom row: Adventitia tissue digitally removed, leaving only collagen stained within the tunica media. (**b**) Quantification of the percent area occupied by collagen within tunic media; data is presented as mean % Area Collagen ± SD. Scale bar = 50 µm. *p < 0.05, ***p < 0.001. One-way ANOVA with Bonferroni t-tests was applied.

Examination of the cross-sectional plane of endothelium-intact carotid artery segments from control rats and stained with H&E revealed a wall thickness of 37.35 ± 1.33 µm. Notably, the carotid artery wall thickness was significantly greater in burned rats at 6 h (43.05 ± 2.77 µm) than at 3 days (33.49 ± 1.13 µm) (*p* < 0.05). We found no other differences in wall thickness between the other time points after burn. These changes were demonstrated temporally without alterations observed at the other time points (Fig. 2). We found no prominent inflammatory response such as infiltration of inflammatory cells in the carotid artery tissue. Using Masson’s trichrome staining, we found that collagen expression significantly increased at day 3 after burn, (*p* < 0.01) and quickly declined thereafter (Fig. 3).

**Figure 4:**
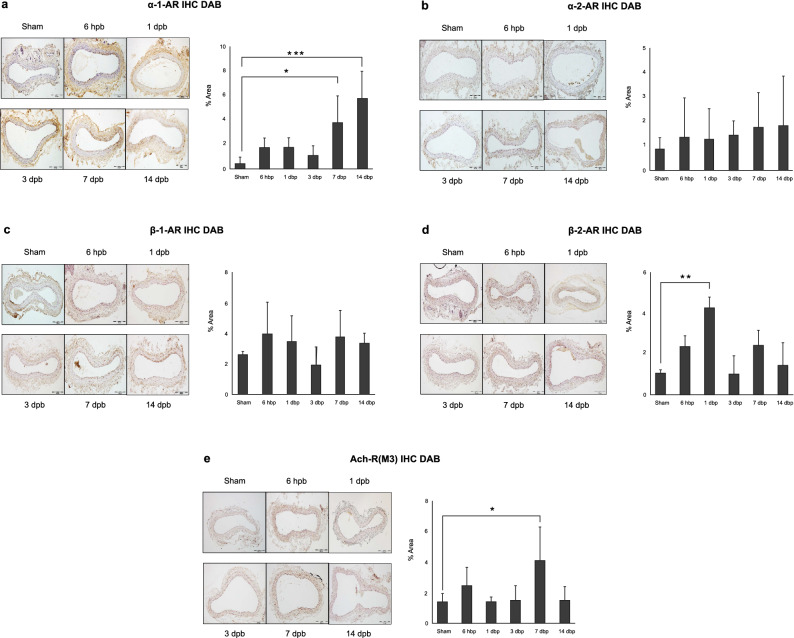
Immunohistochemistry staining and quantification of positive stained expressions of (**a**) α-1a adrenergic receptor (AR), (**b**) α-2a-AR, (**c**) β-1-AR, (**d**) β-2-AR and (**e**) muscarinic acetylcholine M3 receptor in rat carotid artery rings after burn. One-way ANOVA with Bonferroni t-tests was applied Data were presented as mean ± SD. *p < 0.05, **p < 0.01, ***p < 0.001.

Immunohistochemistry (IHC) staining data demonstrated expression of adrenergic receptors (AR) (α1, α2, β1, and β2) and muscarinic acetylcholine M3 receptors changed differently in response to injury over the time (Fig. 4). AR-α1a expression significantly increased within the carotid artery tunica media 7-days after burn (*p* < 0.05). As a negative feedback of inhibitory of norepinephrine releasing, α-2a-AR expression did not significantly change in burned rats. β-1-AR expression also had no change over time after burn; interestingly, functioning as smooth muscle relaxation, a significant elevation of β-2-AR expression within the carotid artery tunica media was observed only at 1-day after burn. Responding to acetylcholine (Ach) stimulation, muscarinic Ach-R (M3) significantly increased expression at 7-days after burn.

### Protein content and signal detections

**Figure 5:**
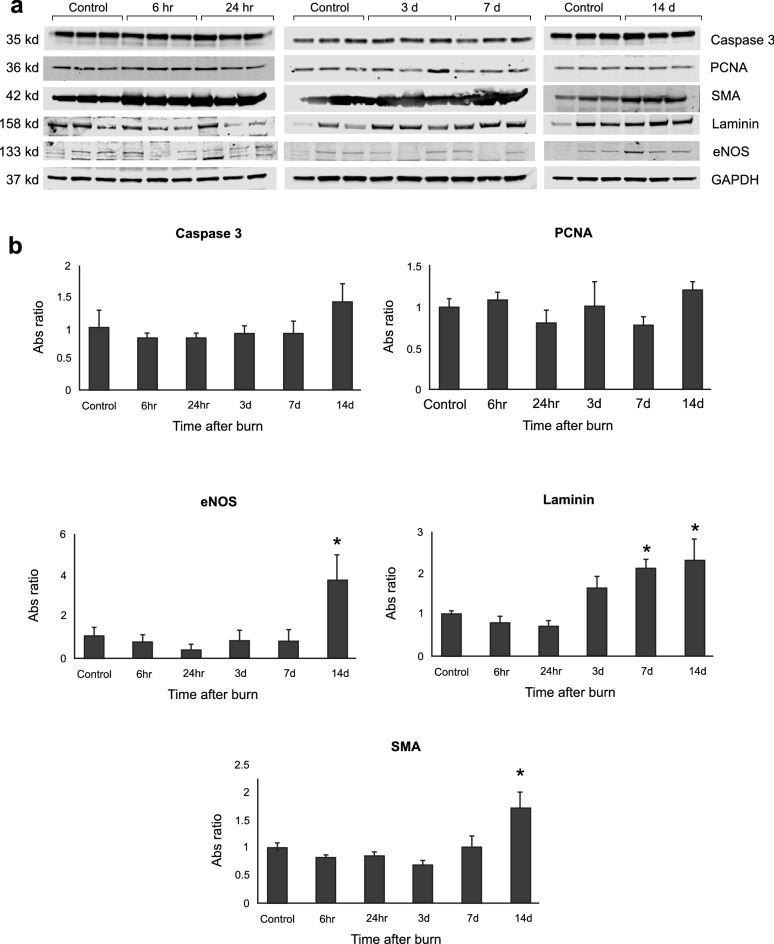
(**a**) Western blot images, and (**b**) Statistical analysis of signals caspase 3, PCNA, eNOS, Laminin, and α-SMA. The absorbance intensity was normalized with internal control GAPDH. *, *p* < 0.05, versus control group, one-way ANOVA with Dunnett’s method was applied. Data were presented as mean ± SEM.

We found no significant changes in carotid artery total protein content in the periods after burn. Tissue homeostasis was evaluated with PCNA and caspase 3 protein expression and normalized to GAPDH. PCNA and caspase 3 expression were unchanged after burn exposure. Laminin is a component of extracellular matrix and functions as an integral part of structural scaffolding. Smooth muscle actin is a “normal” component of VSM and used as a marker of myofibroblast formation during endothelia to mesenchymal transition. We analyzed carotid artery protein using western blot analysis and observed that laminin expression increased over twofold at day 7 and day 14 post-burn and smooth muscle actin increased 1.7-fold at day 14. eNOS protein was detected and observed to increase 3.8-fold at day 14 after burn (*p* < 0.05) (Fig. 5). Raw western blot images were presented in Supplemental Fig. 1.

## Discussion

In this study, we are the first to examine the physiological changes in carotid artery function that occurred in the immediate period after burn as well as the changes that occurred through day 14. These functional changes involved not only responses to several native vasoconstrictors, including NE and AngII, but also the generation and function of NO, a primary mechanism that mediates VSM relaxation and vasodilation. Acutely, the carotid artery smooth muscle exhibits a transitory general increase in vasoconstriction function 24 h after injury, as evidenced by responses to KCl, which occurs via a nonreceptor-mediated mechanism. This could be induced by transit biochemical properties, e.g., changes of elevated lactate or calcium fluctuation. However, we found diminished responses to both vasoconstrictor and vasodilator stimulation at 6 h after burn injury. These changes are likely reversible since tissue protein content and cell number homeostasis were not changed. Following the early acute phase and equilibration after 24 h, endothelial-induced eNOS elevation appears to play an important role in initiating vasodilation at the later time points following burn injury. We also observed increased extracellular matrix protein expression, indicating changes in vascular wall modification following injury with an implied reduction in vasodilation capacity.

In response to injury, epinephrine and norepinephrine regulate cardiovascular tone through activation of alpha^11^ and beta^12^ adrenergic receptors, thereby modulating the distribution of cardiac output and thus peripheral perfusion. Previous studies demonstrated a systemic increase of catecholamine in response to severe burn^13,14^. In the current study, the capacity of the rat carotid artery to generate stresses were decreased 6 h after burn as evidenced by the alteration in the cumulative dose-responses to NE. Thus, either signaling through either VSM alpha- or beta-adrenergic receptors or activation of the VSM mechanisms for contraction have been altered. In 2017, Evans et al.^11^ used a 30% TBSA scald burn rat model to study α-adrenergic receptor agonists in regulating blood pressure from 24 to 168 h after burn. Similar to our study, they observed a transient loss of sensitivity to the α1-adrenergic receptor agonist phenylephrine at 24 h after burn. However, neither their study nor the present one was able to determine which mechanism was responsible for the changes in contractility. Nonetheless, we can conclude from the present study it is not due to increases in NO-mediated relaxation.

**Figure 6:**
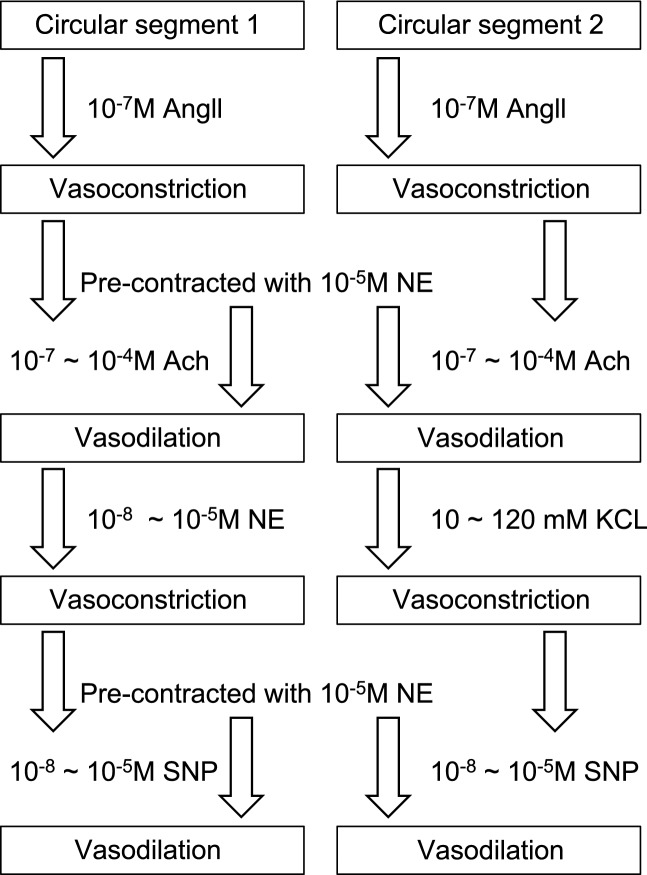
The flow chart of ex vivo carotid artery ring (CA) testing procedures.

In the present study, we examined vascular tone using the paradigm noted in Fig. 6, which follows the protocol we previously used to study carotid artery development and function in early ovine gestation^15^. In that study, we employed endothelium-denuded carotid arteries and demonstrated that changes in VSM function were age and dose dependent. In the current study, we chose to employ endothelium-intact carotid arteries in order to explore the interactions of endothelium and VSM in carotid arteries from burned rats.

Nitric oxide as oxidative stress mediator in vascular disease^16^, plays an important role in mediating tissue and organ homeostasis after burn^17^. Using sodium nitroprusside as a NO donor, we observed that VSM relaxation was diminished from its pre-contraction status 3-days after burn. NO is produced principally from L-arginine via the endothelial enzyme nitric oxide synthase (eNOS)^18^. The vasodilator response to acetylcholine activation of eNOS was absent and recovered 3 days after burn injury. eNOS has the protective function to induce arterial vasodilation, and we found that eNOS protein expression increased 14 days after burn. By comparing the responses to sodium nitroprusside, a NO donor, and acetylcholine, we conclude that eNOS expression compensated vasodilation recovery at the later time point after burn, as the capacity of vasodilation in carotid arteries decreased over the time course.

eNOS expression and activity are controlled by multiple mechanisms. Acetylcholine-induced pulmonary vasodilation and increased eNOS expression during exercise training in a swine model^19^. Further, phosphorylated eNOS changed activities have been studied in trauma ischemic-reperfusion injury^20^. Although we sought to examine changes in two phosphorylated forms of eNOS, we were unable to reliably identify eNOS activity because of the low expression. As for eNOS signal expression, another study in muscle wasting after burn showed that eNOS was not changed in muscle at 3 days after injury^21^. We did not find the change in eNOS at day 3 in our study either. Instead, we found that eNOS increased in 14 days after burn.

After injury, tissue and organ respond and change, then return to the normal equilibrium state. Travers et al.^22^ found that cardiac fibroblasts transform to the myofibroblast phenotype, which contributes to cardiac fibrosis after injury, thus, profibrotic conditions are present after injury, which includes the cardiovascular system^23^. Evidence of long-term effects include identification myocardial fibrosis with a long-term cardiac dysfunction in 18% of severely burned children 2 years after injury^24^. In this study, we found early functional changes in vascular tone, which were associated with histological evidence of early fibrosis. The changes we described here are mostly physiological and functional. Although we did detect evidence of fibrosis with increased laminin and smooth muscle actin expression at later time points, we understood that analyzed sample were homogenates from all three tunica layers of artery. To address this issue, we used Masson’s trichrome staining of arterial tissue directly. The changes of non-receptor contraction and collagen expression in arterial smooth muscle occurs in both histologic and physiological realms occurred coincidentally at 3 days after burn. We speculate that this may indicate initial functional changes which induce consequent vascular remodeling. Further studies will be pursued including interventional evaluations to determine the marginal effects on large arteries after severe burn.

Burn injury impairs the myocardium, which could lead to cardiovascular remodeling. A similar rodent model showed cardiovascular dysfunction through β-AR signaling pathway in the right ventricle^12^. A recent study even showed the mitochondrial signal activated in the heart tissue in burn rats^25^. However, we focused on the smooth muscle contractile in the major artery in response to severe burn. We did not examine the heart remodeling combined with our focus in this study though cardiac changes might certainly guide peripheral changes.

Alterations of pH and lactate affect vascular smooth muscle tone^26,27^, which mainly affect intracellular Ca^2+^ concentrations^28^. Severe burn disrupts biochemical properties in circulating blood with elevated lactate and other electrolytes^29^. We focused this study on the physiological responses mediated through ARs in a peripheral artery following severe burn. We, unfortunately, did not measure blood lactate or pH which may modify vascular contraction after severe burn. Animal studies have showed that lactate and potassium levels were significantly increased 420 min in rats after 30% burn^30^. This could explain that carotid smooth muscle contraction increase with KCL stimulation at the earlier time after burn while immunohistochemistry staining data of elevated α1-adrenergic receptor expression at later time points might partially explain re-elevated response of KCL stimulation through intracellular calcium alterations. Based on our pathological and pathophysiological data, we assumed that different mechanisms dominated smooth muscle contraction over time course after burn. However, the current results could not confirm nor deny this assumption. Further specific study is needed to understand the mechanism of artery smooth muscle contraction in response to severe burn.

Immunohistochemistry showed that adrenergic receptors and muscarinic acetylcholine M3 receptor expression is changed in response to severe burn, which could reflect with vascular ring contractile property in burn rats, although this must be addressed in further directed experiments.

In the current study, we examined changes in vascular function following severe burn injury; however, the intracellular mechanisms were not fully investigated. Some potential mediators of vasoconstriction and vasodilation were not examined in this study, such as histamine-activated histamine receptors (H1/H2)^31^, and another group not examined were natriuretic peptide receptors (NPR)^32^. The role of those receptors should be considered in the future to achieve a full picture.

The observations related to the time after severe burn and change in vascular function are clinically relevant, especially those changes occurring in the first 24 h when we clinically find decreases in vascular responsiveness to both vasoconstrictors and vasodilators. Interestingly, we found similar responses here though these were diminished but not absent. This suggests that vascular tone and peripheral perfusion in the first 24 h after burn injury are not normal but will gradually return toward normal following the initial stabilization and resuscitation. Since arterial function is diminished and not absent, higher doses of vasoactive mediators, in particular vasoconstrictors, may be needed in order to maintain peripheral vascular tone and thus blood pressure which are expected to be transient. This has clinical implications during resuscitation from severe burn when hypotension is not uncommon. These observations also suggest that if normovolemia is a primary goal during initial resuscitation, the temporary use of adrenergic agents may be indicated until normal vascular sensitivity returns and facilitates the maintenance of systemic blood pressure and peripheral perfusion.

## Conclusions

In conclusion, we showed that changes in carotid artery sensitivity to vasoconstrictors and the vasodilator NO occur soon after a severe burn and that these changes are time and dose-dependent. Moreover, vascular tone is regulated by the interactions of several mediators, including calcium and G-protein coupled receptor pathways, which drive vasoconstriction and vasodilation. These VSM mediators are regulated by different receptors, which alternately oppose vascular physiological activities and competing regulation mechanisms. These changes in vascular functional are associated with parallel changes in vascular remodeling and signs of fibrosis.

## Methods

Forty-three adult male Sprague Dawley rats (~ 300 g; Charles River Laboratories, Wilmington, MA) were used in this study. The animals were acclimated for 7 days before the experimental procedure. The procedures followed NIH guidelines and were approved by the IACUC at UT Southwestern Medical Center (UTSW) where the studies were performed. The study was carried out in compliance with the ARRIVE guidelines. Animals were housed individually with free access to commercial rat chow and tap water.

### Burn procedure

Thirty-four rats received a 40% TBSA scald burn following an established procedure^33^ with minor modifications of Walker-Mason model^34^. The rodent model has been proven to induce hyperinflammation response^35^ and systemic metabolic response^36^. Under anesthesia with 2–4% isoflurane inhalation, rats were shaved and secured in a constructed template device to expose the back. The shaved skin exposed through the template was immersed in 100 °C water for 12 s to produce full-thickness cutaneous burns over 40% TBSA. Animals were then resuscitated with intraperitoneal Lactated Ringer’s solution (4 ml per kg body weight per percentage burn area), as well as subcutaneous buprenorphine 0.5 mg/kg for analgesia. Nine sham-burned rats received the same procedure without hot water immersion and intraperitoneal resuscitation. Animals were randomly allocated and euthanized with overdose of isoflurane inhalation following thoracotomy exsanguination between 6 h and 14 days after injury.

### Tissue harvesting

Common carotid arteries were isolated by blunt dissection from the adjacent sternocleidomastoid muscle and an approximately 0.6 cm length was excised from both sides proximal to the internal/external carotid artery bifurcation. The intact carotid artery was rinsed with room temperature PBS to remove clots. The right carotid artery was divided transversely into two segments; one frozen in liquid nitrogen and stored at − 80 °C and the other fixed in 10% neutral formalin buffer. The left carotid artery was also divided transversely into two pieces and transferred to an organ bath for relaxation-contraction studies.

### Carotid smooth muscle contractile function

Carotid artery preparation and functional measurements have previously been described^15^. Briefly, endothelium intact carotid artery segments were cut into two 3 mm-length rings and studied in duplicate. Each artery ring was immersed in physiological salt solution buffer (120.5 mM NaCl, 4.8 mM KCl, 1.2 mM MgSO_4_, 1.2 mM NaH_2_PO_4_, 20.4 mM NaHCO_3_, 1.6 mM CaCl_2_, 10 mM dextrose, and 1.0 mM pyruvate) for 30 min at room temperature. Following equilibration, each ring was placed on a stirrup attached to a force transducer in a 25 ml volume organ bath and stretched with 2-g loads followed by 65 mM KCl treatment until the optimal equilibration length (L_o_) was reached. All subsequent studies were performed at L_o_.

Artery segments were systematically exposed to vasoconstrictors and vasodilators in order to describe the properties for each vasoactive compound (Fig. 6). Initially, all segments were exposed to 10^–7^ M angiotensin II (Ang II). We then examined the responses to cumulative doses of acetylcholine (Ach; 10^–7^ M to 10^–4^ M) after precontraction with 10^–5^ M NE. This was followed by either a cumulative dose response to NE (10^–8^ M to 10^–5^ M) or to KCl (10 to 120 mM). Following equilibration, each ring was then precontracted with 10^–5^ M NE and cumulative relaxation responses to sodium nitroprusside (SNP; 10^–8^ to 10^–5^ M). Artery rings were gently flushed with fresh physiological salt solution at each end of stimulation and the baseline stress reset before the next drug exposure. Arterial responses were recorded in grams of force at L_o_ on an electronic data-acquisition system (ACQuire, Gould Systems, Valley View, OH) and data are presented as stress in Newtons/m^2^. All chemical agents were obtained from Sigma Aldrich.

### Tissue histology, immunohistochemistry staining, and quantification

Fixed arterial segments were embedded in paraffin, following 5 µm sectioning and stained with hematoxylin and eosin^15^. Under 10 × magnification of histological images, and arterial wall thickness were measured by blinded observers (RG, RC) at 10 × magnification calibrated with a standard slide micrometer. Wall thickness was measured from the *tunica interna* (the single endothelium layer) to *tunica media* (external elastic lamina) and acquired in 4 directions for each segment.

#### Trichrome staining

7 µm of paraffin embedded tissue sections were deparaffinized followed the rehydration procedure. Tissues were then incubated in Bouin’s solution overnight at room temperature. The next day, sections were stained to visualize tissue collagen by Masson’s trichrome following the manufacture instruction [Cat. No. KTTRBPT, American Master Tech, Statlab, Lodi, CA, USA] and counterstained in hematoxylin. Standard digital photomicrographs for four quadrants of each transverse carotid artery section were captured at 400 × magnification (Olympus BX45 pathology microscope with DP73 color camera) utilizing cellSens software (cellSens standard 1.7 with Manual Acquisition Process for cellSens Standard 1.11, Olympus, Center Valley, PA, USA). Total collagen was then analyzed for every image by a validated color thresholding method^37^ using ImageJ software^38^; color threshold for collagen remained the same for every image analyzed. The tunica media, the area between the internal and external elastic laminae, was isolated by digitally removing the tunica adventitia and any remaining tissue. Once thresholded, the percent area occupied by collagen within the tunica media was quantified by masked particle analysis. Percent area measurements were calculated for each of the high-power images per tissue section (N = 4 per sample).

#### Immunohistochemistry staining

7 µm of paraffin embedded tissue section was deparaffinized and rehydrated by standard protocol. To expose target proteins, antigen retrieval was performed using 10 mM sodium citrate (pH 6.0) heated to approximately 175 °C for 30 min. Following antigen retrieval, tissues were incubated in 3% H_2_O_2_-methanol for 30 min at room temperature to quench endogenous peroxidase activity, washed in PBS, and then blocked in 1% goat serum for 1 h. Tissues were then probed for specific primary antibodies: Rabbit polyclonal adrenergic receptor antibodies with dilution factors including α-1a (cat#PA1-047, 1:200), α-2a (cat# PA1-048, 1:75), β1 (cat#BS-0498R, 1:275), and β2 (cat#13096-1-AP, 1:275) were purchased from Fisher Scientific. Anti-Muscarinic Acetylcholine Receptor M3/CHRM3 antibody was purchased from Abcam (ab87199, 1:150). Tissues were incubated in primary antibody diluted in DAKO (Agilent, Santa Clara, CA, USA) background reducing antibody diluent overnight at 4 °C. The next morning, sections were warmed to room temperature, washed in PBS, and incubated in biotinylated secondary anti-rabbit (IgG) antibody for 30 min (VECTASTAIN Elite ABC-HRP Kit, cat# PK6101). Sections were then washed three times in PBS and then an Avadin/Biotin-HRP solution was applied for 30 min at room temperature per manufacturer’s instruction. Next, 3-3′-diaminobenzadine (DAB, Vector Laboratories, cat#SK-4105) substrate was applied to each section for 2.5 min to produce brown precipitate that indicated positive primary antibody staining. Sections were then counterstained in hematoxylin to identify purple nuclei. Brightfield photomicrographs were obtained at 100 × magnification (Olympus BX45) for each carotid artery section under the same light conditions (intensity and exposure). Each image was analyzed for positive staining through ImageJ software using the validated IHC toolbox quantification method for DAB staining^39^. For each target receptor, percent positively stained area within only the tunica intima and tunica media was quantified by the method described above (trichrome staining).

### Protein extraction and immunoblotting

Frozen arterial tissue was weighed and approximately 20 mg of tissue was homogenized in T-PER lysis buffer (Thermofisher, Waltham, MA). Tissue protein content was measured with Bradford protein assay (BioRad, Hercules, CA) using FlUOstar OPTIMA Microplate Reader (BMG Labtech, Cary, NC). Tissue protein (20 µg) were analyzed by SDS-PAGE electrophoresis and western immunoblotting for signal protein detection with established methods^40^. Antibodies, including eNOS, phosphor-eNOS (S1177) and phosphor-eNOS (Thr495), were from ThermoFisher. Caspase 3 and proliferating cell nuclear antigen (PCNA), and glyceraldehyde 3-phosphate dehydrogenase (GAPDH) were from Cell Signaling Tech (Danvers, MA), and α-smooth muscle actin (SMA), laminin was from Abcam (Cambridge, MA). GAPDH was used as the control protein on all immunoblots.

### Statistical analysis

The number of animals was 9, 7, 7, 7, 7 and 6 for the control, 6 h, 1 day, 3 days, 7 days, and 14 days groups, respectively. The collective data from sham animals were presented as the control group.

Two-way analysis of variance (ANOVA), one-way ANOVA, with post hoc tests were used where appropriate. SigmaPlot 14.0 software (Systat Software, San Jose, CA) was used to determine statistical significance, *p* < 0.05. Global curve fitting analysis was applied to describe dose response. Statistical significance was set at significance level α < 0.05, the probability of rejecting the null hypothesis .

### Conference presentation

Data was presented at the Annual Shock Society Conference on June 6–9, 2020.

## Supplementary Information

Supplementary Information.
