# Supplementary material for: Carotid smooth muscle contractility changes after severe burn
Source: Sci Rep. 2021 Sep 10;11:18094. doi: 10.1038/s41598-021-97732-3 (PMC8433376; doi:10.1038/s41598-021-97732-3)
Supplement: Supplementary file 1 — Supplementary Information. [file 41598_2021_97732_MOESM1_ESM.pdf]

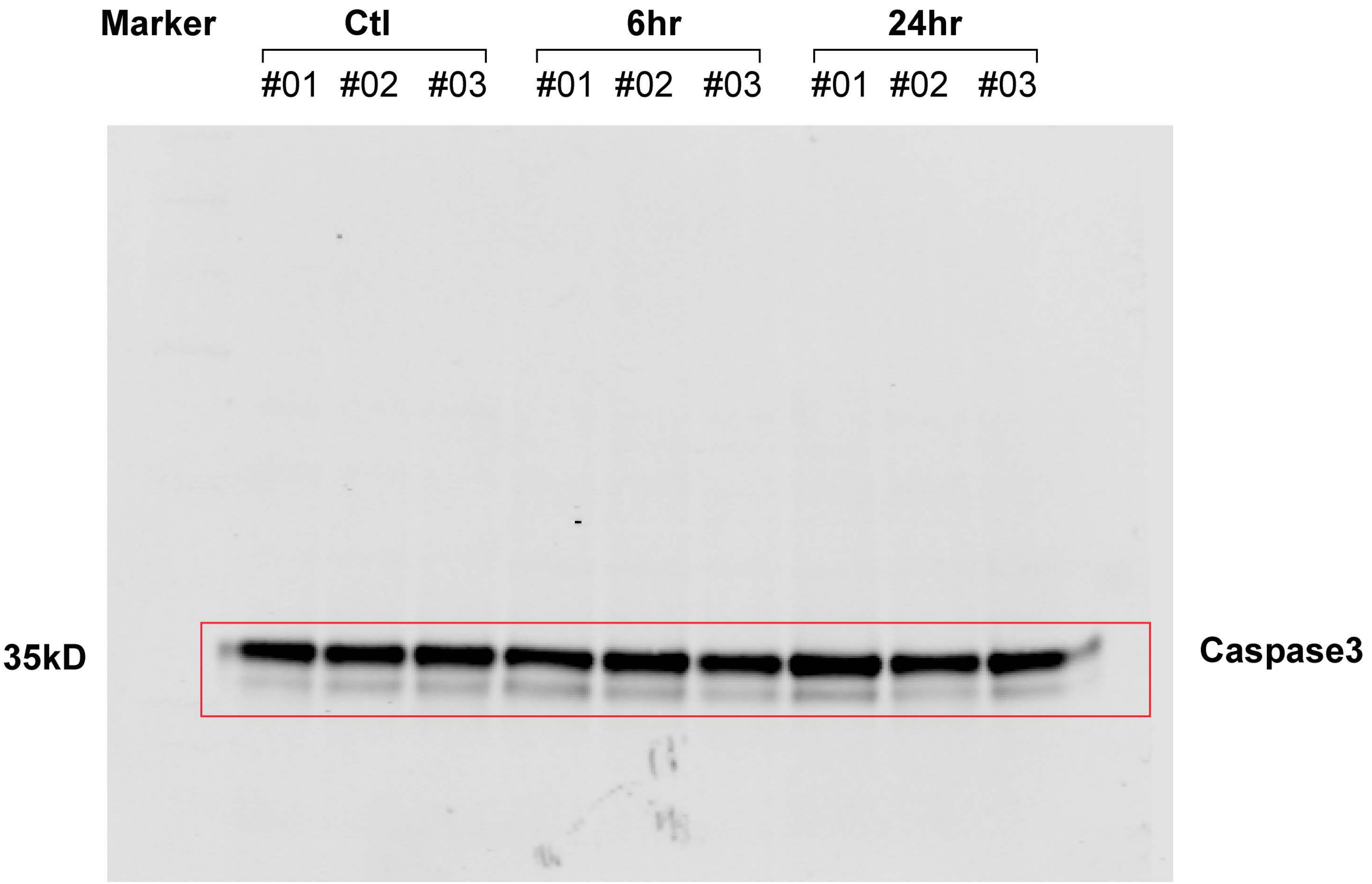

Supplemental figure 1:
Western blot raw image of caspase 3 expressions in rat carotid tissue at 6 and 24 hours after burn. The 1st lane is the molecular weight marker, and the following lanes with 20ug of protein lysate from control (Ctl), 6 hours (6hr), and 24 hours (24hr) groups (n=3/each group).
Three samples at the control group were repeatedly applied in all raw blots following the same order.
A red box circled caspase 3 protein band at the range of 35 kD.

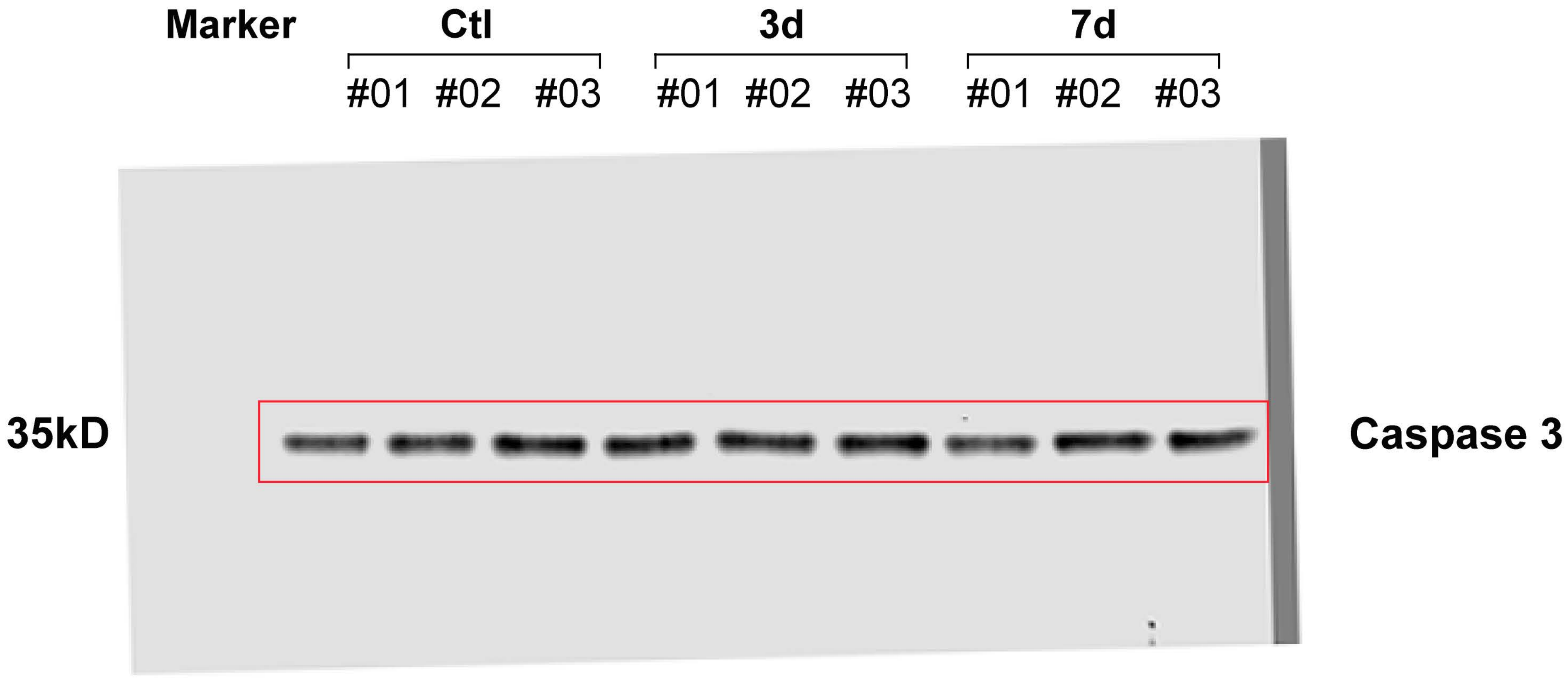

Supplemental figure 2:
Western blot raw image of caspase 3 expressions in rat carotid tissue at 3 and 7 days after burn. The 1st lane is the molecular weight marker, and the following lanes with 20ug of protein lysate from control(Ctl), 3days (3d) and 7 days (7d) groups (n=3/each group).
Three samples at control group were repeatedly applied in all raw blots following the same order.
A red box circled caspase 3 protein band at the range of 35 kD.

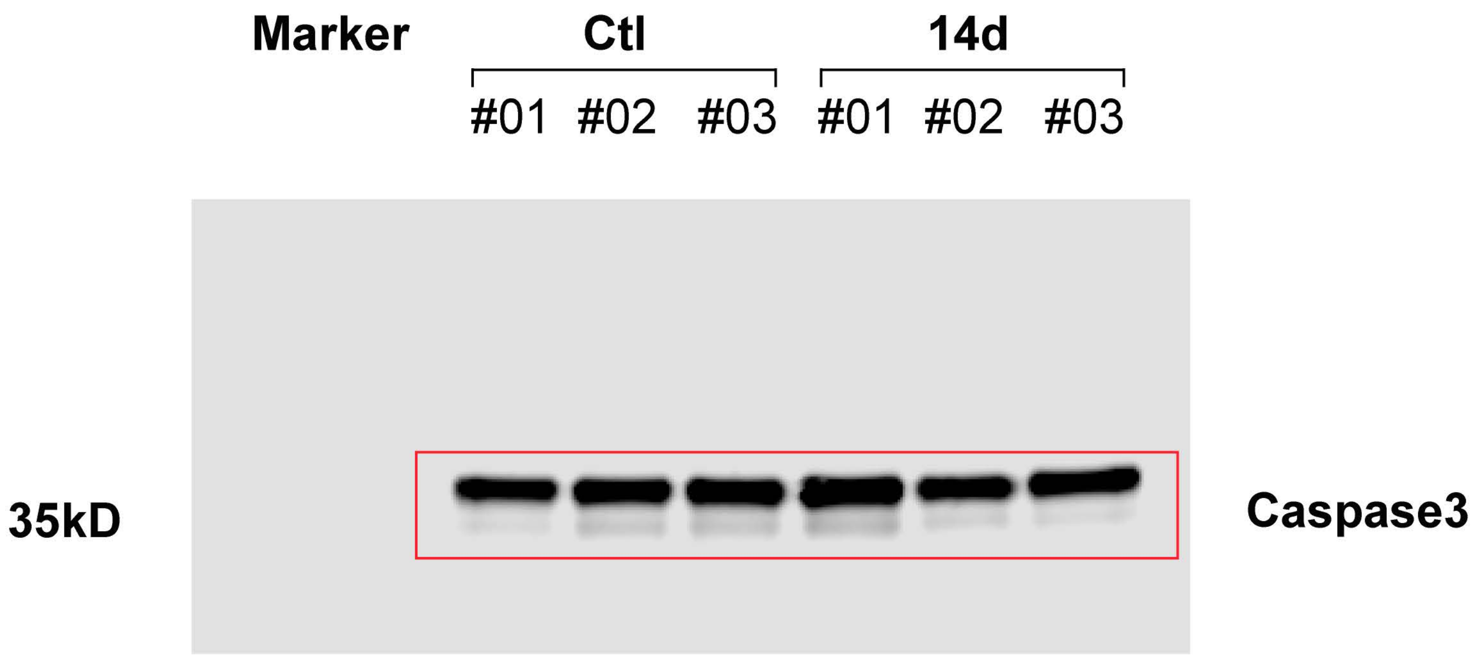

Supplemental figure 3:
Western blot raw image of caspase 3 expressions in rat carotid tissue at 14 days after burn. The 1st two lanes are the molecular weight markers, and the following lanes with 20ug of protein lysate from control (Ctl) and 14 days (7d) groups (n=3/each group).
Three samples at control group were repeatedly applied in all raw blots following the same order.
A red box circled caspase 3 protein band at the range of 35 kD.

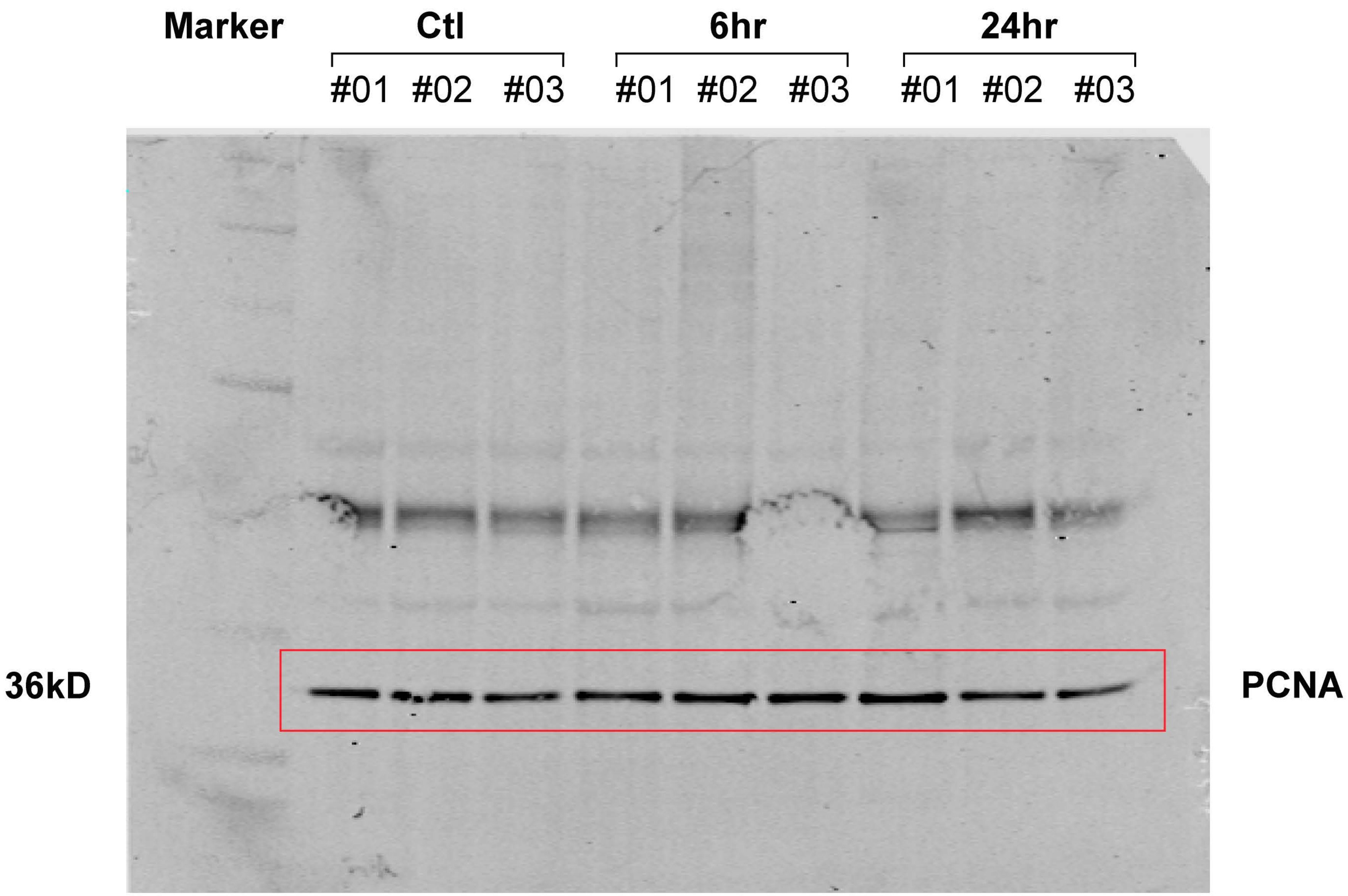

Supplemental figure 4:

Western blot raw image of PCNA expressions in rat carotid tissue at 6 and 24 hours after burn. The 1st lane is the molecular weight marker, and the following lanes with 20ug of protein lysate from control (Ctl), 6 hours (6hr), and 24 hours (24hr) groups (n=3/each group).
Three samples at control group were repeatedly applied in all raw blots following the same order.
A red box circled PCNA protein band at the range of 36 kD.

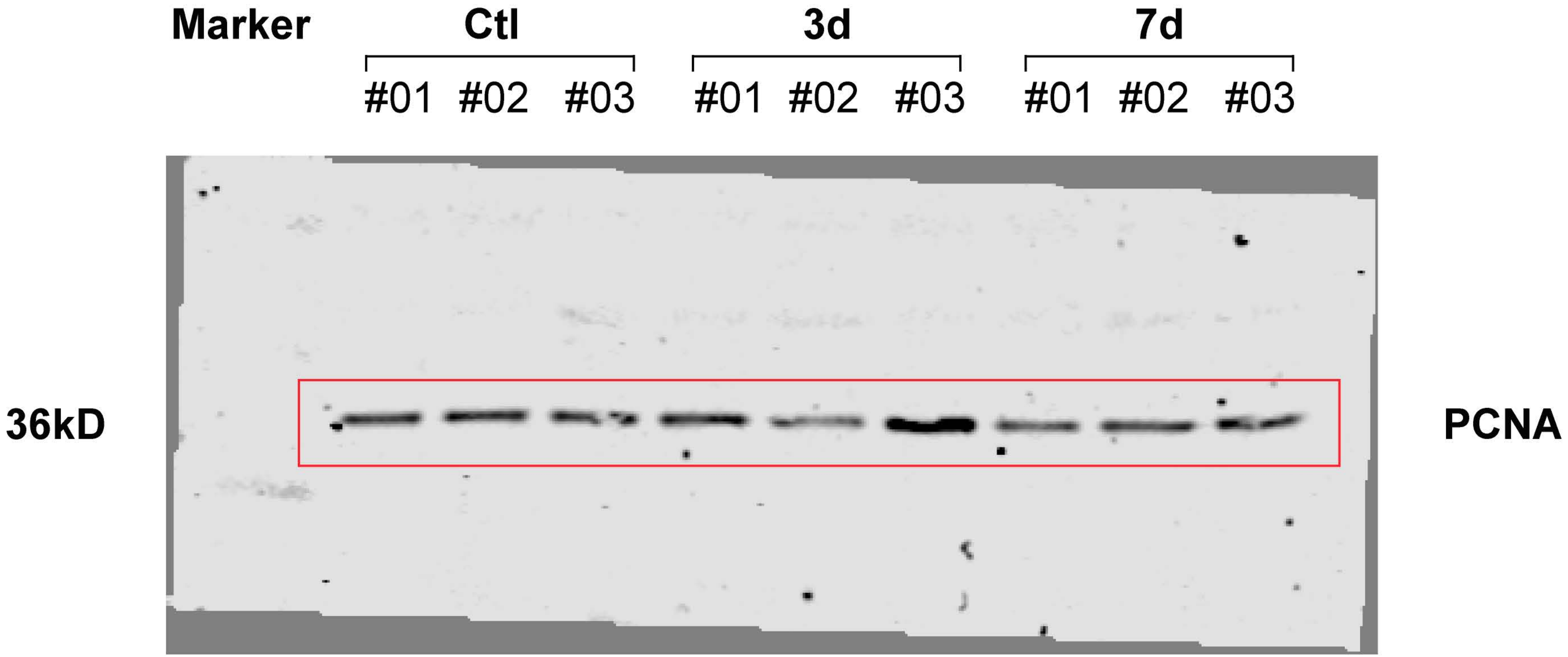

Supplemental figure 5:

Western blot raw image of PCNA expressions in rat carotid tissue at 3 and 7 days after burn. The 1st lane is the molecular weight marker, and the following lanes with 20ug of protein lysate from control (Ctl), 3 days (3d), and 7 days (7d) groups (n=3/each group).
Three samples at control group were repeatedly applied in all raw blots following the same order.
A red box circled PCNA protein band at the range of 36 kD.

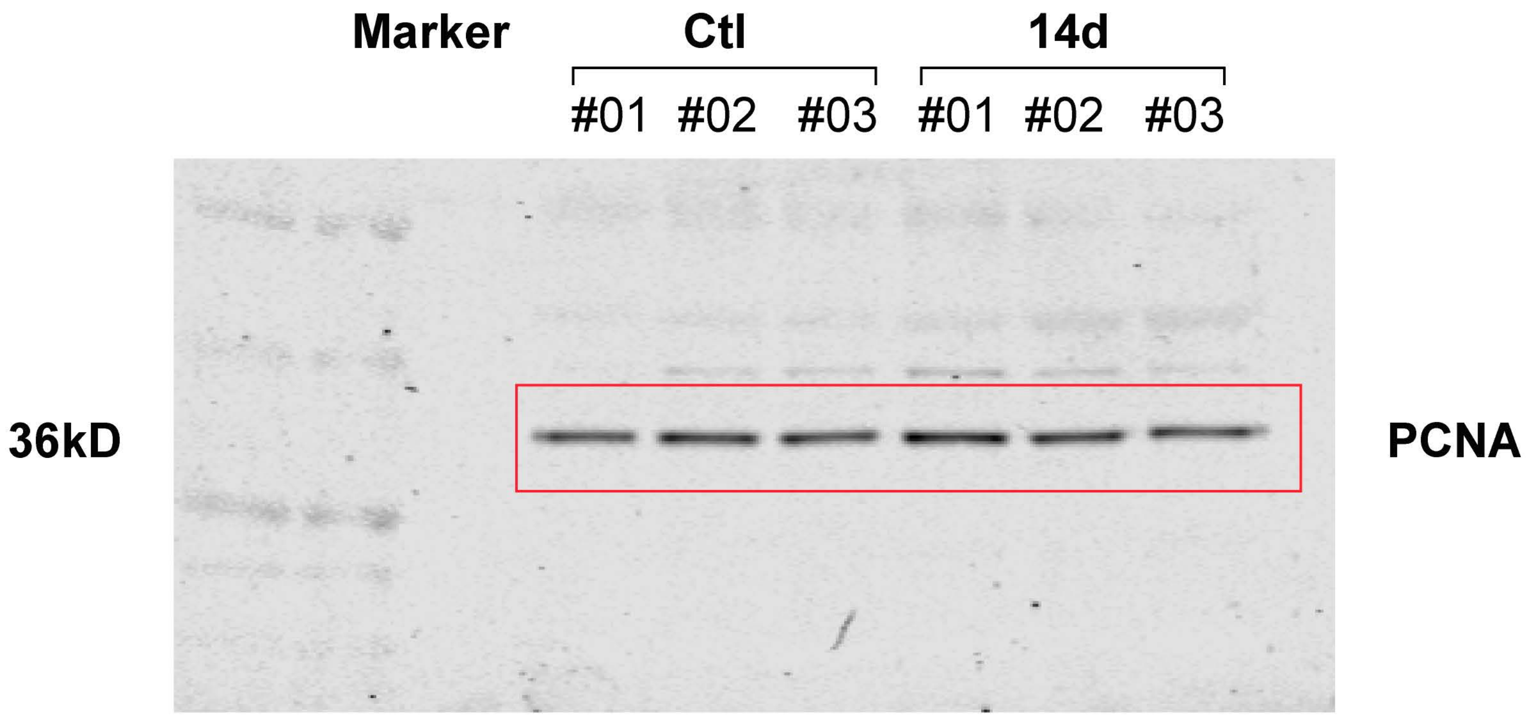

Supplemental figure 6:
Western blot raw image of PCNA expressions in rat carotid tissue at 14 days after burn. The 1st two lanes are the molecular weight markers, and the following lanes with 20ug of protein lysate from control(Ctl) and 14 days (7d) groups (n=3/each group).
Three samples at control group were repeatedly applied in all raw blots following the same order.
A red box circled PCNA protein band at the range of 36 kD.

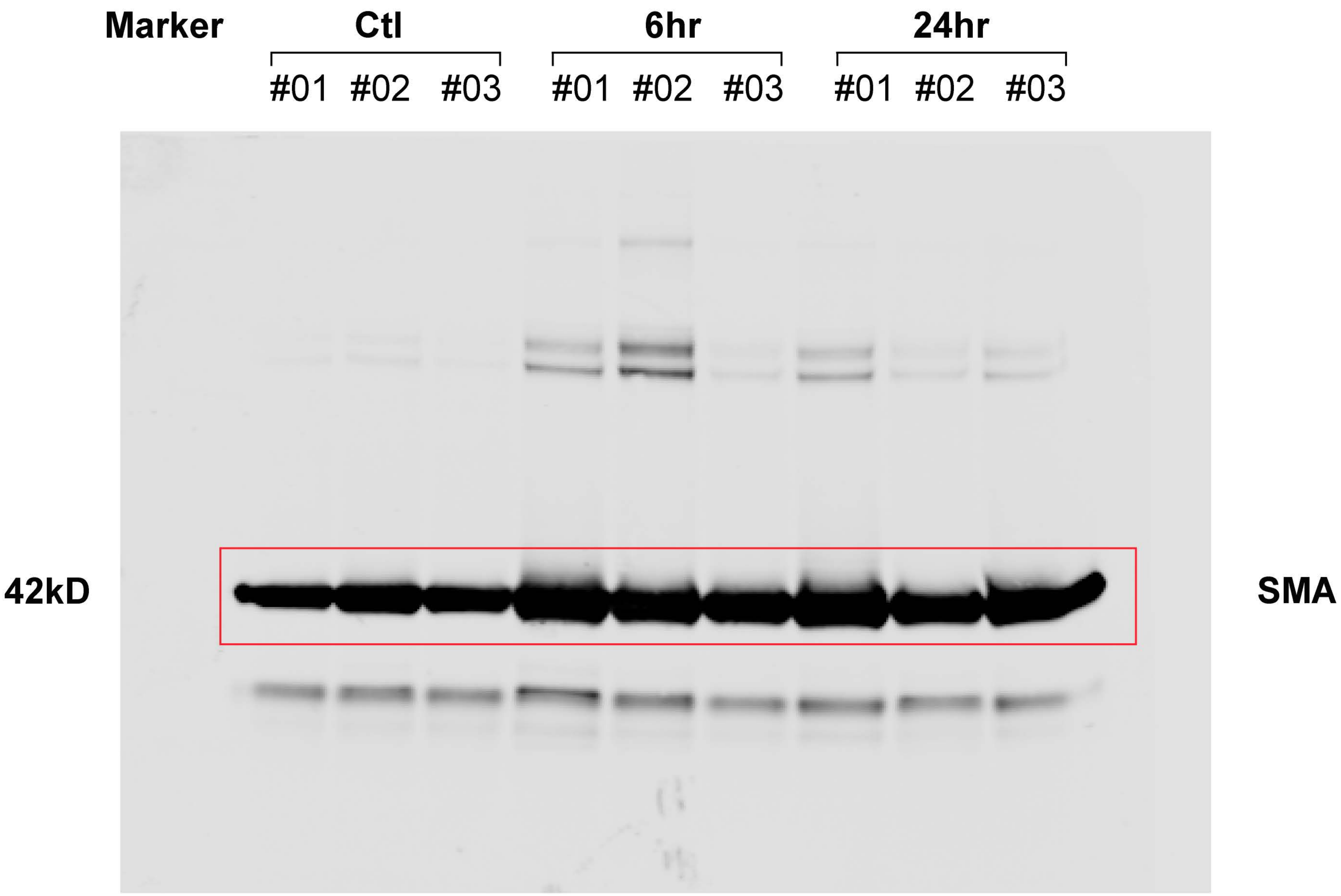

Supplemental figure 7:

Western blot raw image of SMA expressions in rat carotid tissue at 6 and 24 hours after burn. The 1st lane is the molecular weight marker, and the following lanes with 20ug of protein lysate from control (Ctl), 6 hours (6hr), and 24 hours (24hr) groups (n=3/each group).
Three samples at control group were repeatedly applied in all raw blots following the same order.
A red box circled SMA protein band at the range of 42 kD.

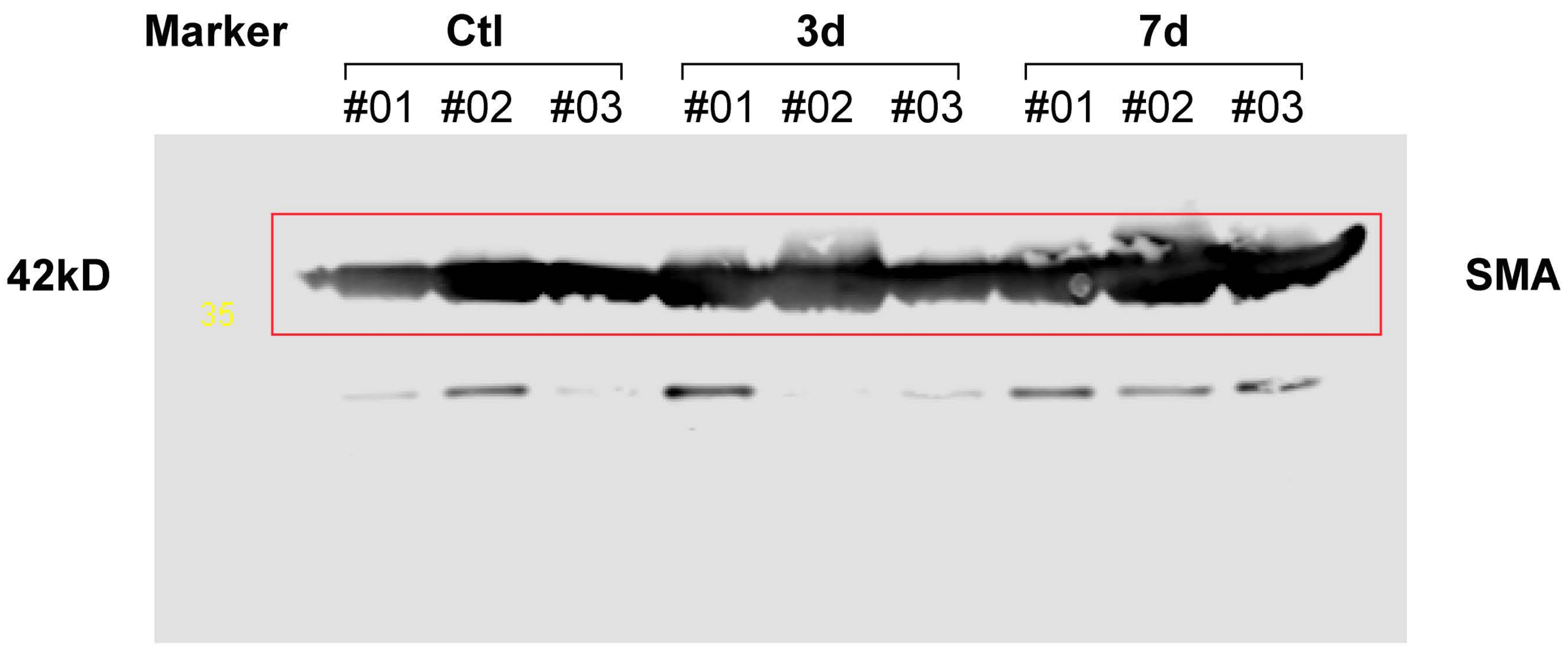

Supplemental figure 8:
Western blot raw image of SMA expressions in rat carotid tissue at 3 and 7 days after burn. The 1st lane is the molecular weight marker, and the following lanes with 20ug of protein lysate from control (Ctl), 3 days (3d), and 7 days (7d) groups (n=3/each group).
Three samples at control group were repeatedly applied in all raw blots following the same order.
A red box circled SMA protein band at the range of 42 kD.

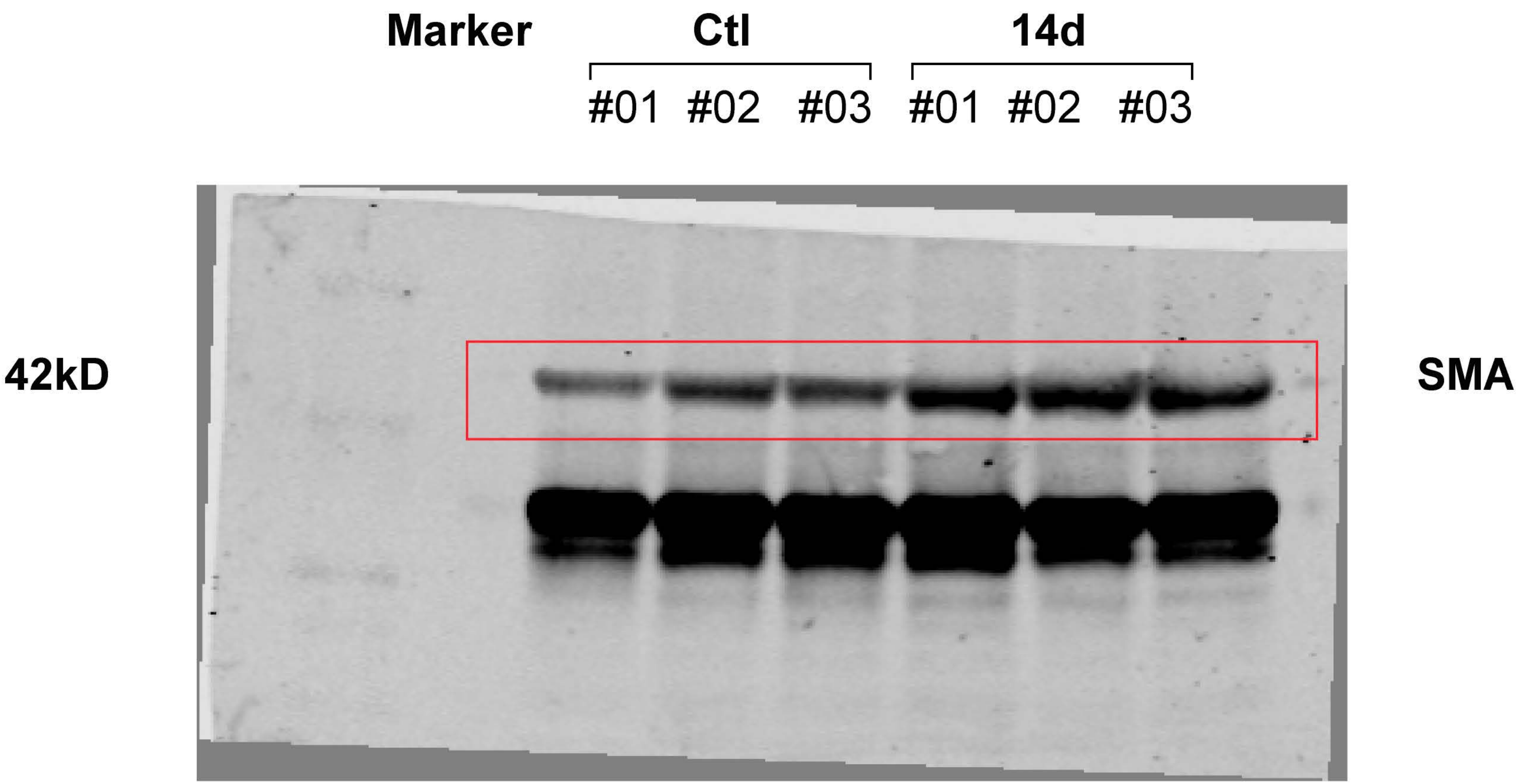

Supplemental figure 9:

Western blot raw image of SMA expressions in rat carotid tissue at 14 days after burn. The 1st two lanes are the molecular weight markers, and the following lanes with 20ug of protein lysate from control (Ctl) and 14 days (7d) groups (n=3/each group).
Three samples at control group were repeatedly applied in all raw blots following the same order.
A red box circled SMA protein band at the range of 42 kD.

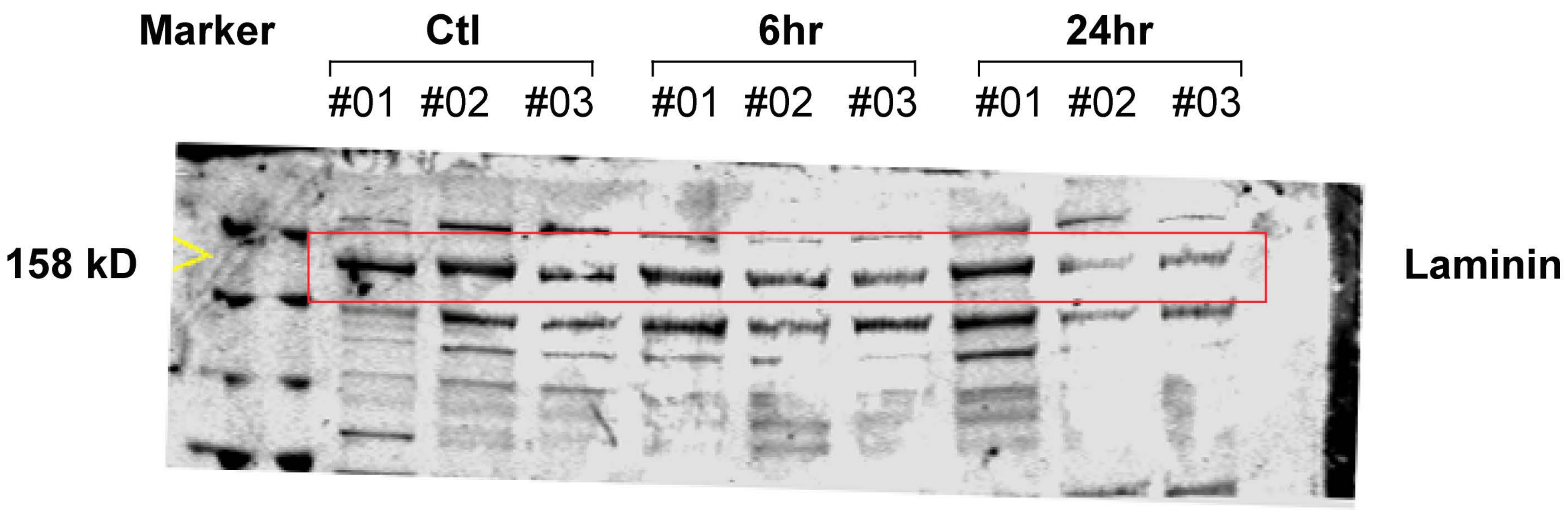

Supplemental figure 10:

Western blot raw image of laminin expressions in rat carotid tissue at 6 and 24 hours after burn. The 1st lane is the molecular weight marker, and the following lanes with 20ug of protein lysate from control (Ctl), 6 hours (6hr), and 24 hours (24hr) groups (n=3/each group).
Three samples at control group were repeatedly applied in all raw blots following the same order.
A red box circled laminin protein band at the range of 158 kD.

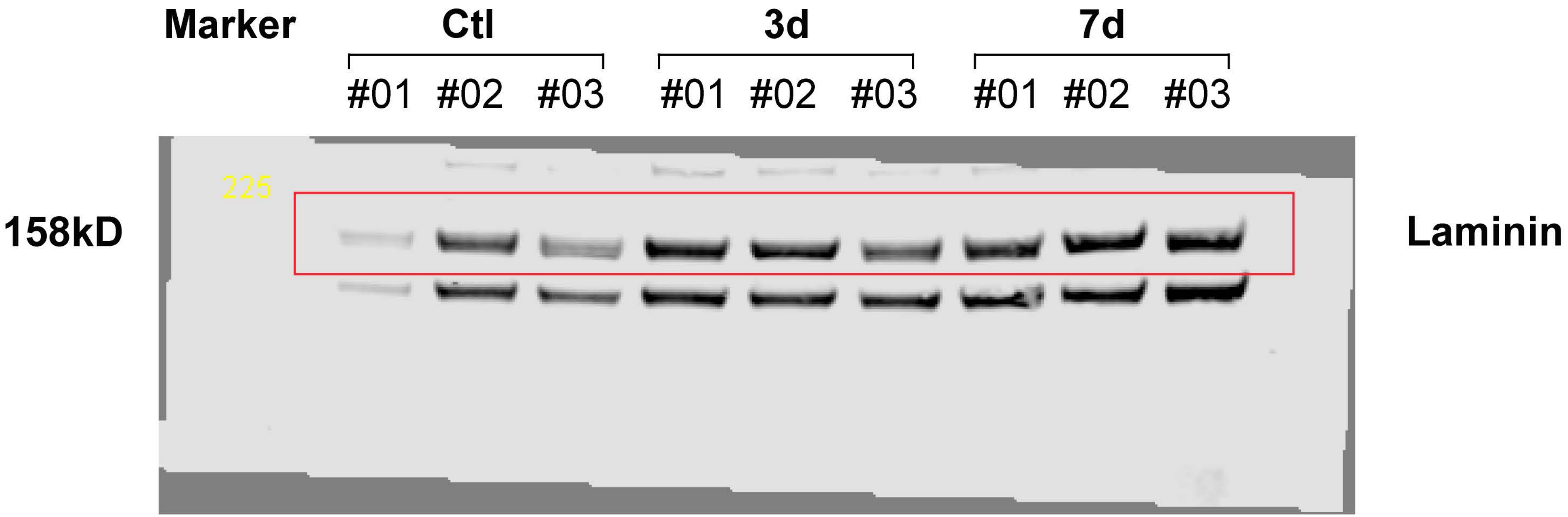

Supplemental figure 11:
Western blot raw image of laminin expressions in rat carotid tissue at 3 and 7 days after burn. The 1st lane is the molecular weight marker, and the following lanes with 20ug of protein lysate from control (Ctl), 3 days (3d), and 7 days (7d) groups (n=3/each group).
Three samples at control group were repeatedly applied in all raw blots following the same order.
A red box circled laminin protein band at the range of 158 kD.

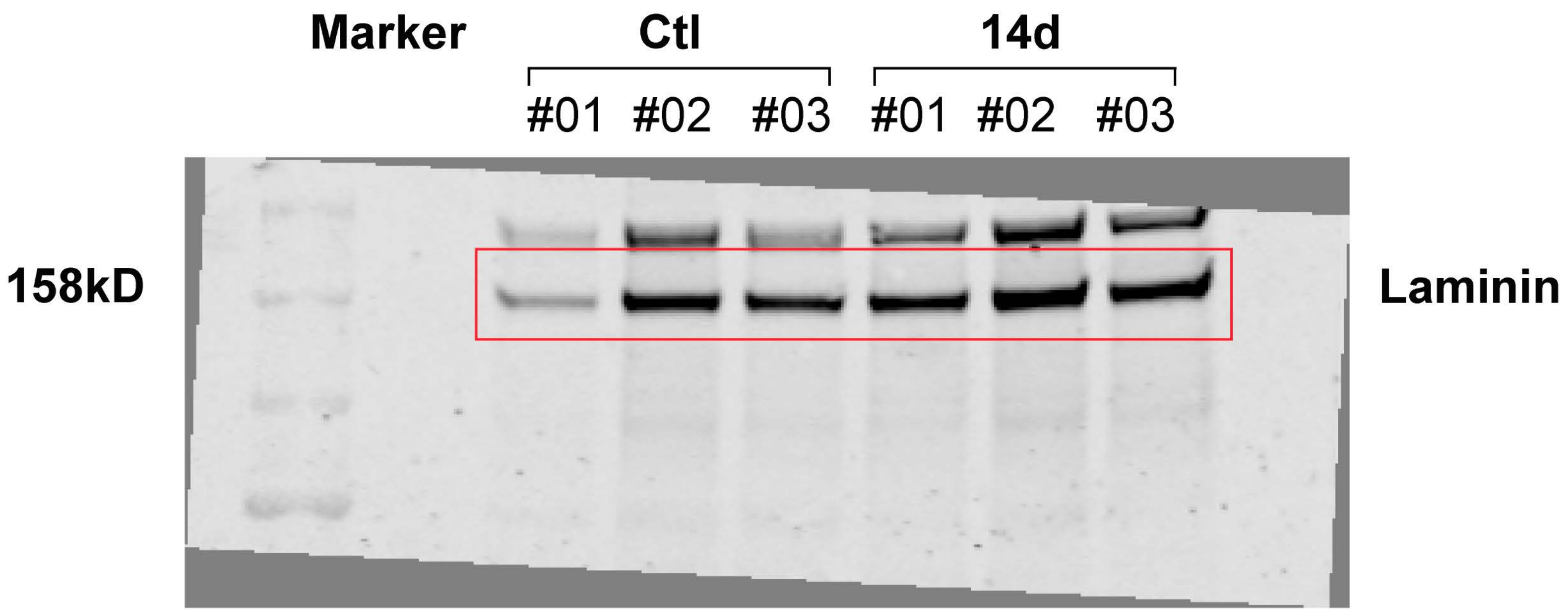

Supplemental figure 12:

Western blot raw image of laminin expressions in rat carotid tissue at 14 days after burn. The 1st two lanes are the molecular weight markers, and the following lanes with 20ug of protein lysate from control(Ctl) and 14 days (7d) groups (n=3/each group).
Three samples at control group were repeatedly applied in all raw blots following the same order.
A red box circled laminin protein band at the range of 158 kD.

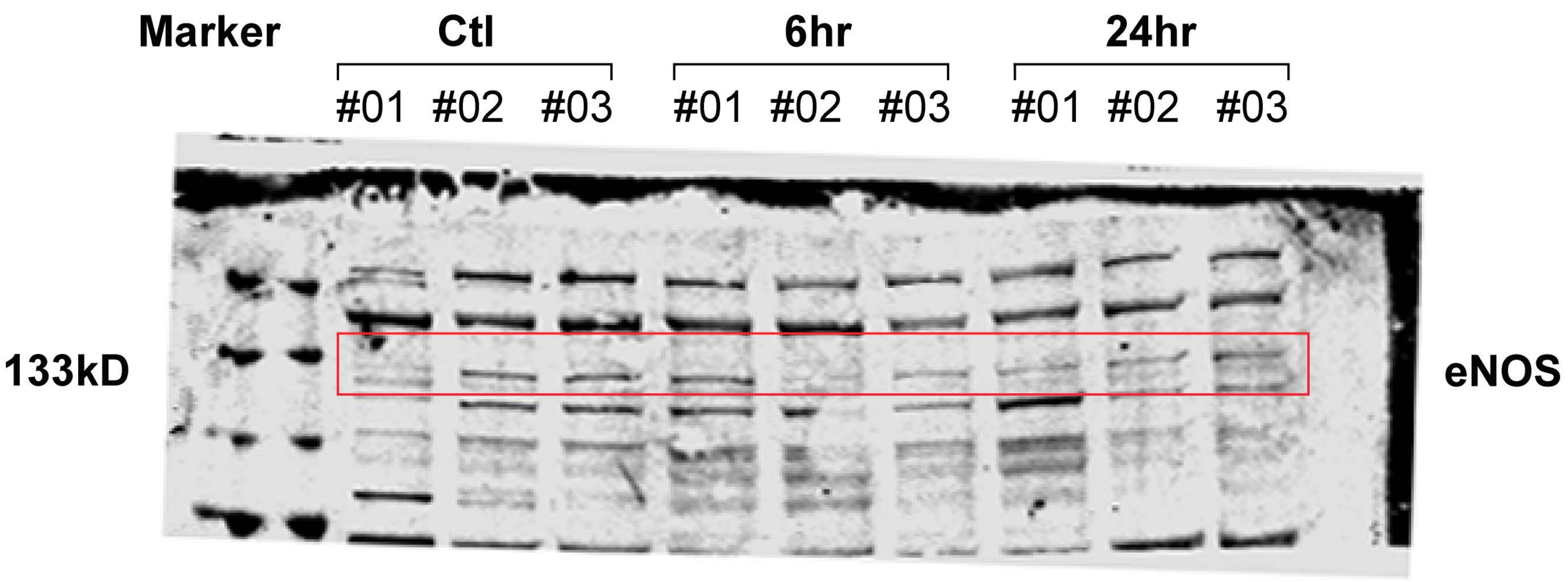

Supplemental figure 13:
Western blot raw image of eNOS expressions in rat carotid tissue at 6 and 24 hours after burn. The 1st lane is the molecular weight marker, and the following lanes with 20ug of protein lysate from control (Ctl), 6hours (6hr), and 24hours (24hr) groups (n=3/each group).
Three samples at control group were repeatedly applied in all raw blots following the same order.
A red box circled eNOS protein band at the range of 133 kD.

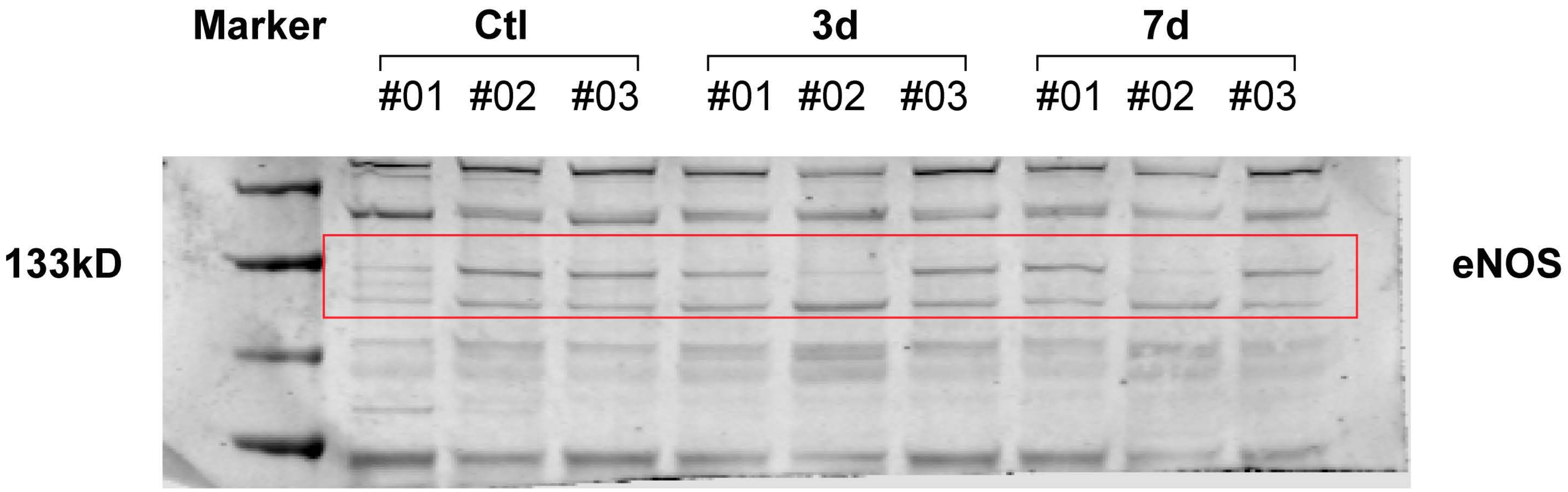

Supplemental figure 14:

Western blot raw image of eNOS expressions in rat carotid tissue at 3 and 7 days after burn. The 1st lane is the molecular weight marker, and the following lanes with 20ug of protein lysate from control(Ctl), 3days (3d) and 7 days (7d) groups (n=3/each group).
Three samples at control group were repeatedly applied in all raw blots following the same order.
A red box circled eNOS protein band at the range of 133 kD.

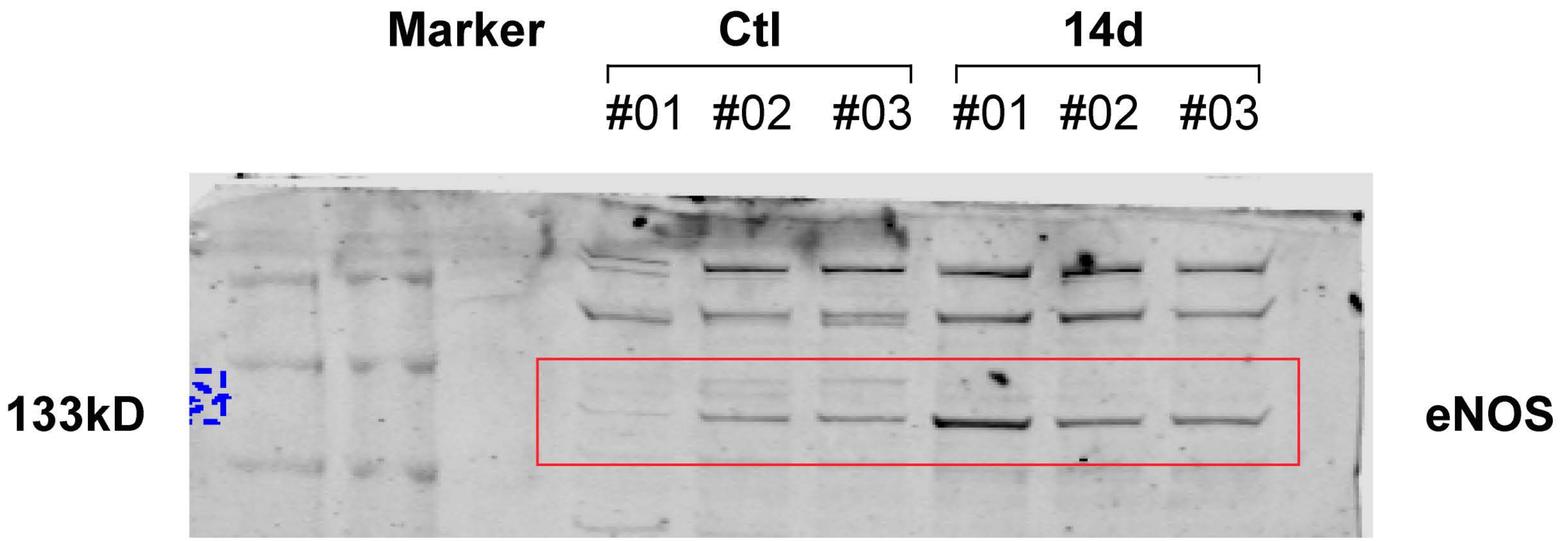

Supplemental figure 15:

Western blot raw image of eNOS expressions in rat carotid tissue at 14 days after burn. The 1st two lanes are the molecular weight markers, and the following lanes with 20ug of protein lysate from control (Ctl) and 14 days (7d) groups (n=3/each group).

Three samples at control group were repeatedly applied in all raw blots following the same order.

A red box circled eNOS protein band at the range of 133 kD.

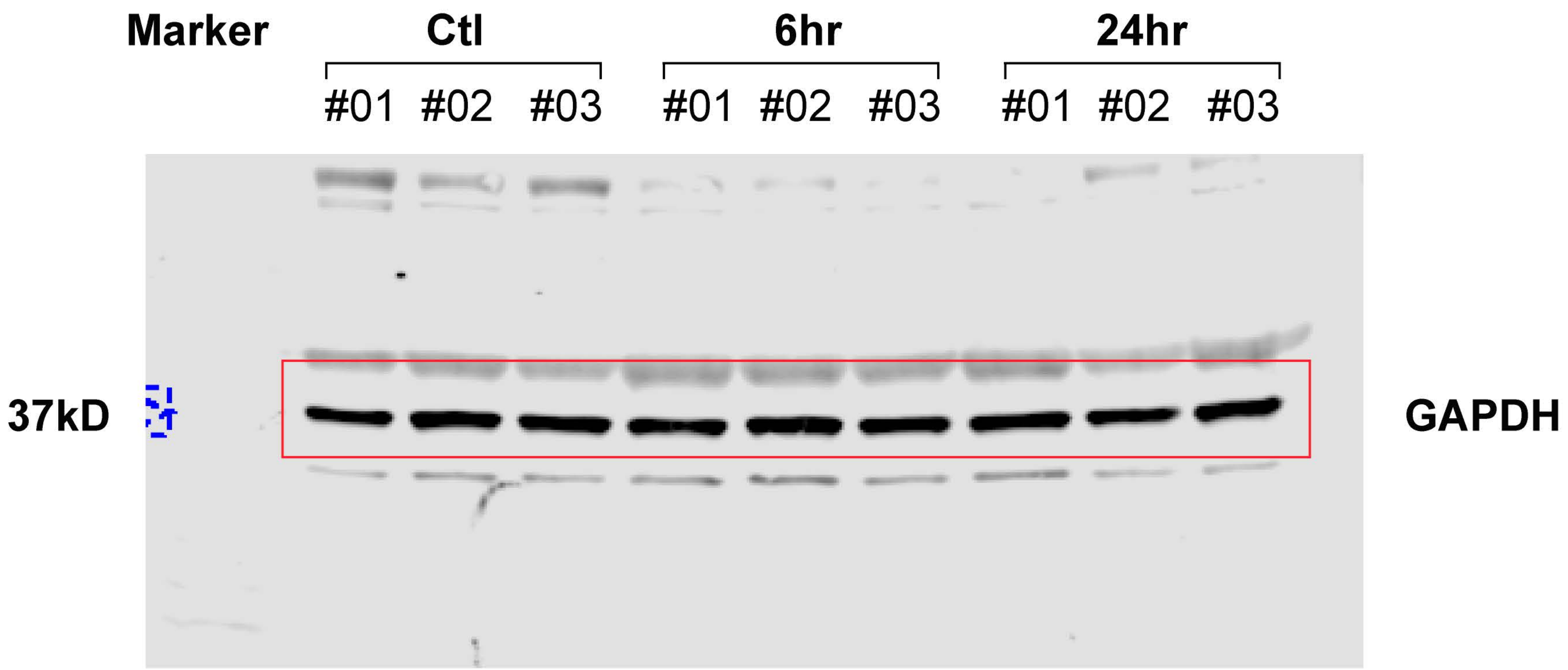

Supplemental figure 16:

Western blot raw image of GAPDH expressions in rat carotid tissue at 6 and 24 hours after burn. The 1st lane is the molecular weight marker, and the following lanes with 20ug of protein lysate from control (Ctl), 6 hours (6hr), and 24 hours (24hr) groups (n=3/each group).
Three samples at control group were repeatedly applied in all raw blots following the same order.
A red box circled GAPDH protein band at the range of 37 kD.

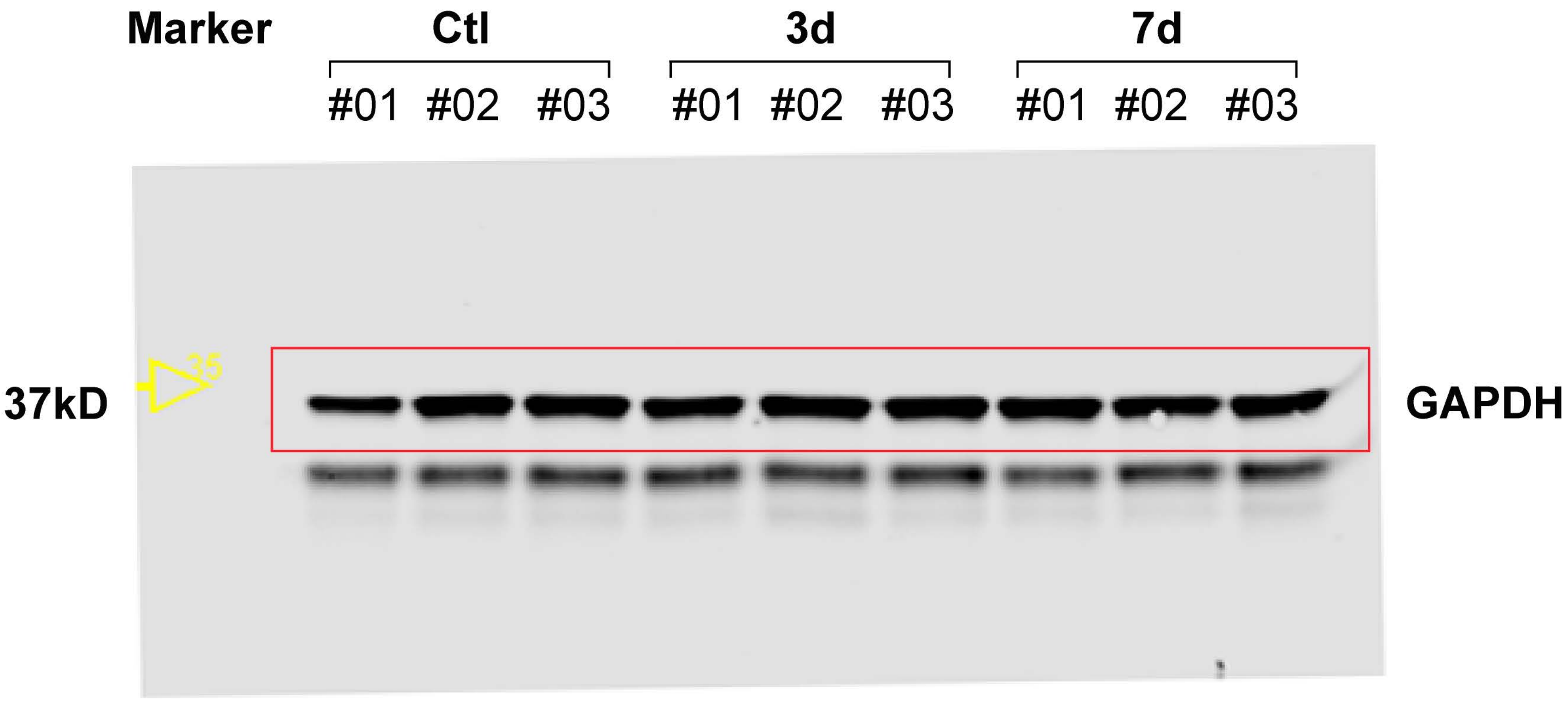

Supplemental figure 17:
Western blot raw image of GAPDH expressions in rat carotid tissue at 3 and 7 days after burn. The 1st lane is the molecular weight marker, and the following lanes with 20ug of protein lysate from control (Ctl), 3 days (3d), and 7 days (7d) groups (n=3/each group).
Three samples at control group were repeatedly applied in all raw blots following the same order.
A red box circled GAPDH protein band at the range of 37 kD.

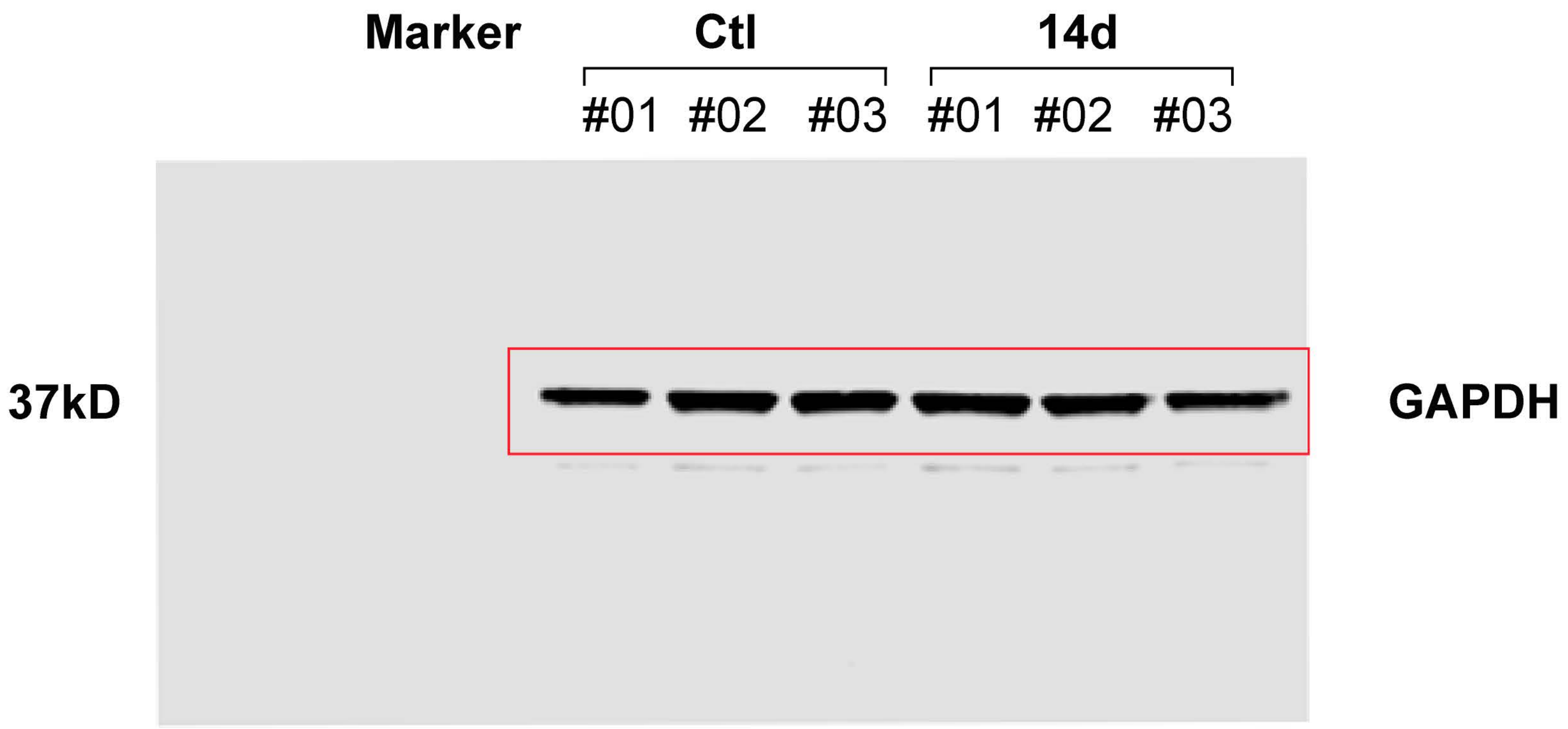

Supplemental figure 18:

Western blot raw image of GAPDH expressions in rat carotid tissue at 14 days after burn. The 1st two lanes are the molecular weight markers, and the following lanes with 20ug of protein lysate from control(Ctl) and 14 days (7d) groups (n=3/each group).

Three samples at control group were repeatedly applied in all raw blots following the same order.

A red box circled GAPDH protein band at the range of 37 kD.
